# Effects of a Synbiotic Formula on Functional Bowel Disorders and Gut Microbiota Profile during Long-Term Home Enteral Nutrition (LTHEN): A Pilot Study

**DOI:** 10.3390/nu13010087

**Published:** 2020-12-29

**Authors:** Valentina D’Onofrio, Federica Del Chierico, Paola Belci, Pamela Vernocchi, Andrea Quagliariello, Sofia Reddel, Giorgia Conta, Maria Vittoria Mancino, Maurizio Fadda, Maria Carmine Scigliano, Roberta Morelli, Antonella De Francesco, Fabio Guagnini, Filippo Fassio, Rosalba Galletti, Lorenza Putignani

**Affiliations:** 1 S.C. Dietetica e Nutrizione Clinica, AOU Città della Salute e della Scienza di Torino, 10126 Torino, Italy; donofriovalentina.vdo@gmail.com (V.D.); paola.belci@gmail.com (P.B.); mmancino@cittadellasalute.to.it (M.V.M.); mfadda@cittadellasalute.to.it (M.F.); mscigliano@cittadellasalute.to.it (M.C.S.); rmorelli2@cittadellasalute.to.it (R.M.); adefrancesco@cittadellasalute.to.it (A.D.F.); rgalletti@cittadellasalute.to.it (R.G.); 2 Multimodal Laboratory Medicine Research Area, Unit of Human Microbiome, Bambino Gesù Children’s Hospital, IRCCS, 00165 Rome, Italy; federica.delchierico@opbg.net (F.D.C.); pamela.vernocchi@opbg.net (P.V.); andrea.quagliariello@opbg.net (A.Q.); sofia.reddel@opbg.net (S.R.); 3 Department of Chemistry, Sapienza University of Rome, 00185 Rome, Italy; giorgia.conta@uniroma1.it; 4 NMR-Based Metabolomics Laboratory, Sapienza University of Rome, 00185 Rome, Italy; 5 Allergy Therapeutics Italia, Milan, Italy e GE Healthcare, 20019 Milan, Italy; fabio.guagnini@allergytherapeutics.com; 6 Allergy and Clinical Immunology Unit, San Giovanni di Dio Hospital, Azienda USL Toscana Centro, 50143 Florence, Italy; fassio.filippo@gmail.com; 7 Department of Diagnostic and Laboratory Medicine, Unit of Parasitology and Multimodal Laboratory Medicine Research Area, Unit of Human Microbiome, Bambino Gesù Children’s Hospital, IRCCS, 00147 Rome, Italy

**Keywords:** bedridden patients, long-term home enteral nutrition (LTHEN), synbiotics, constipation, diarrhoea, gut microbiota, dysbiosis

## Abstract

Long-term enteral nutrition (LTEN) can induce gut microbiota (GM) dysbiosis and gastrointestinal related symptoms, such as constipation or diarrhoea. To date, the treatment of constipation is based on the use of laxatives and prebiotics. Only recently have probiotics and synbiotics been considered, the latter modulating the GM and regulating intestinal functions. This randomized open-label intervention study evaluated the effects of synbiotic treatment on the GM profile, its functional activity and on intestinal functions in long-term home EN (LTHEN) patients. Twenty LTHEN patients were recruited to take enteral formula plus one sachet/day of synbiotic (intervention group, IG) or enteral formula (control group, CG) for four months and evaluated for constipation, stool consistency, and GM and metabolite profiles. In IG patients, statistically significant reduction of constipation and increase of stool consistency were observed after four months (T_1_), compared to CG subjects. GM ecology analyses revealed a decrease in the microbial diversity of both IC and CG groups. Biodiversity increased at T_1_ for 5/11 IG patients and *Methanobrevibacter* was identified as the biomarker correlated to the richness increase. Moreover, the increase of short chain fatty acids and the reduction of harmful molecules have been correlated to synbiotic administration. Synbiotics improve constipation symptoms and influences *Methanobrevibacter* growth in LTHEN patients.

## 1. Introduction

The gut microbiota (GM), composed of 10^14^ microbes inhabiting the human intestine, is a complex ecological community that influences physiology and disease susceptibilities through its collective metabolic activities and host interactions [1]. The GM protects against pathogens, extracts nutrients and energy from diet, and contributes to normal immune function [2]. Dysbiosis, disruption of the normal balance between GM and host, has been associated with obesity, malnutrition, inflammatory bowel diseases (IBD), neurological disorders, cancer, and other gastrointestinal (GI) and extra-intestinal diseases [3,4,5,6,7,8].

There is vast gut microbial diversity and it is highly variable, both over time and across human populations. In particular, there is a gradient in bacteria concentration across the body, along the GI tract and colon itself, and from the low concentrations transiting from the ileum to the cecum [9].

The colon is the only substantial contributor to the total bacterial population, while the stomach and small intestine (duodenum and jejunum) make negligible contributions owing to the relatively low pH of the stomach and the fast flow of the content through the stomach and the small intestine [10].

The Firmicutes and Bacteroidetes phyla have been identified as the major bacterial groups present in the mammalian intestine and lactobacilli, anaerobic streptococci, and members of the *Bacteroides* spp. have been identified as resident species of the normal adult human intestine [9,11]. Regional differences in the colic region, with a specific spatial organization, have also been highlighted, especially regarding Lactobacilli, whose presence varies depending on the subject, probably based on specific nutrients and pH [11,12].

Bacterial diversity depends on both the host genetic profile and environmental factors. Diet contents and quantity play a major role in shaping the human GM composition and function [13]. In fact, a diet rich in nonglycemic carbohydrates (so-called dietary fibre) facilitates the presence of fermentative bacteria such as *Bifidobacterium* spp. and *Lactobacillus* spp., while a diet rich in fats and meat increases the presence of putrefactive bacteria, leading to the formation of carcinogenic substances [14]. Enteral nutrition (EN) is a safe nutrition therapy given via a tube or stoma into the GI tract, distal to the oral cavity, to patients whose oral intake of food and fluids is impossible or inadequate for reaching their defined target. A tube can be inserted via the nose (nasogastric, nasojejunal, or naso-post-pyloric tube feeding) or via a stoma that is inserted into the stomach by percutaneous endoscopic gastrostomy, percutaneous radiological gastrostomy, or into the jejunum [15].

Long-term EN, especially when used exclusively, can thus be expected to induce changes in the GM. In patients on total long-term EN, there is indeed dysbiosis characterized by a decrease in the healthy microbial communities and an increase in potentially pathogenic bacteria, with a drop in luminal anaerobic bacteria and increase in aerobic bacteria [16,17]. Symptomatic outcomes of these GM changes include diarrhoea, the most frequent complication of early enteral feeding, while in patients receiving enteral nutrition, constipation is the most frequently reported gastrointestinal problem [17,18,19,20]. In the literature, several studies on the role, tolerability, and efficacy of fibre-supplemented enteral formulae in the impairment of bowel function are available, although doubts remain about it [21]. In particular, the use of fibre-containing enteral formulae has been associated to the risk of mesenteric ischemia [22]. Moreover, it has been reported that the EN supplemented by fibre could cause intestinal mucosal injury and interfere with nutrient absorption due to the increases demands of blood flow by enterocytes [23].

A systematic review demonstrates the significant clinical benefits of fibre-supplemented enteral feeds in patients suffering from diarrhoea, with a positive trend also observed for patients with constipation [24]. Moreover, other studies investigated the beneficial effects of probiotics in the management of constipation and diarrhoea, despite the absence of full consistency [25].

To date, the treatment of long-term EN-dependent constipation is based on the use of laxative and prebiotics [26], and more recently on probiotics and synbiotics, which represent a promising alternative in restoring intestinal eubiosis [25,27]. However, there is scarceness of literature data about the functional effect of synbiotics on the GM in presence of constipation.

The aim of our study was to investigate the effects of a synbiotic on the modification of GM and intestinal function in long-term home enteral nutrition (LTHEN) patients.

## 2. Materials and Methods 

### 2.1. Study Design and Randomization

This was a randomized open-label intervention study for four months in duration. The randomization list was drawn up by an operator who did not take part in the study. A number was assigned to each patient. The procedure was completely concealed to researchers. The study was not blinded. Indeed, the doctors and dieticians who evaluated the questionnaires and the laboratory personnel who analysed the blood and stool samples were not blinded to the participants’ group assignment.

### 2.2. Ethical Aspects

The current version of the Declaration of Helsinki (2013) was a reference for the ethical aspects of this study and was respected by all participants in this research. Legal tutors and/or participants gave written informed consent to participate in the study. The study protocol was approved by the Ethics Committee of the Città della Salute e della Scienza Hospital of Turin (18 October 2018, prot. n. 0103801).

### 2.3. Recruitment of Participants

Participants were recruited from home enteral nutrition group of Dietetic and Clinical Nutrition of the Città della Salute e della Scienza of Turin, from January 2015 to January 2017. All the enrolled patients were suffering from neurological disorders without digestive disorders before home EN. Inclusion criteria were long-term EN (≥2 years) and no use of antibiotics within three months before the study. Exclusion criteria were active neoplastic disease and progressive neurological diseases (e.g., amyotrophic lateral sclerosis and multiple sclerosis). All patients received fibre-enriched enteral formula. Protein and fluid requirements were considered to be 1 g/Kg/day and 30–40 mL/kg/day, respectively. During the study period, laxative and prokinetic therapy was discontinued.

### 2.4. Intervention 

Twenty bedridden, long-term home EN (LTHEN) patients (average age: 75.2 ± 4.3 years) were randomized to enteral formula plus one sachet/day of synbiotics for four months of study (intervention group, IG, *n* = 11; 6 M/5 F) or enteral formula only for four months (control group, CG, *n* = 9; 5 M/4 F). Allergy Therapeutics Italia (Milan, Italy) provided the synbiotic product, namely Syngut. Each sachet contained 10^9^ colony-forming units (CFU) of *Lactobacillus acidophilus* W22, 3.33 × 10^6^ CFU of *Bifidobacterium lactis* W51, 3.33 × 10^6^ CFU of *Lactobacillus plantarum* W21, 3.33 × 10^6^ CFU of *Lactococcus lactis* W21, and 0.375 g of Inulin. 

### 2.5. Clinical and Nutritional Assessment and Sample Collection

The nutritional evaluations were performed at baseline (T_0_) and after four months (T_1_). During visits, nutritional assessment, enteral feeding, and tolerance of enriched-fibre formula were evaluated. Moreover, the “Constipation Scoring System” (CSS) questionnaire [28] was administered to all patients and/or legal tutors.

Blood samples were collected after overnight enteral feeding. All laboratory measurements were centralized according to manufacturer’s protocols. 

Stool samples were collected at home by patients or legal tutors and delivered to the Dietetic and Clinical Nutrition Department of Città della Salute e della Scienza for a Bristol Stool Chart (BSC) assay [29]. An aliquot of each sample was sent on dry ice to the Human Microbiome Unit of Bambino Gesù Children’s Hospital and the Research Institute of Rome for 16S rRNA-targeted metagenomic analysis. 

Statistical analysis of nutritional and clinical data was performed by Mann–Whitney and Student’s *t*-tests. In both CSS and BCS histograms, median values of each score were reported. 

### 2.6. Gut Microbiota Analysis

Bacterial DNA was extracted from faecal samples using a QIAmp Fast DNA Stool mini kit (Qiagen, Hilden, Germany) according to the manufacturer’s instructions. Amplification and sequencing of V3–V4 16S rRNA gene (≈460 bp) was carried out following MiSeq rRNA Amplicon Sequencing protocol (Illumina, San Diego, CA, USA) on the Illumina MiSeqTM platform according to the procedures described in Romani et al. [30].

Raw data were trimmed for their quality Phred score (>25Q), read length and chimera presence were analysed using the Qiime v1.9 pipeline [31]. Then, the obtained sequences were organized into operational taxonomic units (OTUs) with a 97% clustering threshold of pairwise identity. For each OTU cluster, one representative sequence was aligned using PyNAST v.0.1. [32], then used for multiple sequence alignment (MSA) against the Greengenes 13_08 database with a 97% similarity for bacterial sequences [33]. Finally, the MSA was used to infer a phylogenetic tree [34]. The OTU table, phylogenetic tree, and metadata were used to perform further ecological analysis using the Vegan and Phyloseq packages of R software [35] and to compare taxa’s relative abundance through the Mann–Whitney test and Linear discriminant analysis Effect Size (LEfSe) analysis [36].

### 2.7. MG Data Open Access Repository

All Illumina sequencing raw reads and associated metadata are available at NCBI: Bioprojects PRJNA664661.

### 2.8. Volatile Organic Compounds (VOCs)

Detection of volatile organic compounds (VOCs) was performed on faecal samples by gas chromatography–mass spectrometry solid phase microextraction (GC-MS/SPME) according to Vernocchi et al. [8], by using the carboxen-polydimethylsiloxane coated fibre (CAR-PDMS; 85 μm) and the manual solid-phase microextraction (SPME) holder (Supelco Inc., Bellefonte, PA, USA). The SPME fibre was exposed to each sample for 45 min. The fibre was then inserted into the GC injection port (10 min) for sample desorption and the GC-MS analyses carried out on an Agilent Technologies 7890B GC, coupled to a 5977A mass selective detector operating in electron impact mode (ionization voltage 70 eV), within a 1 mm quartz liner fitted system, equipped with an Agilent DB-HeavyWaX capillary column (60 m lenght, 0.25 mm ID, 0.25 µm). Run conditions were previously reported in Botticelli et al. [37]. The chromatograms were managed by integration and identification with comparison of the fragment pattern with those in the mass spectral NIST library (version 2.2, NIST 14MS database; National Institute of Standards and Technology, Rockville, MD, USA) and literature [38], followed by manual visual inspection. Quantitative data compounds were expressed as parts per million (ppm) (mg/kg) obtained by interpolation of the relative areas vs. internal standard (IS) area. Metabolomic profiles were analysed by univariate (e.g., Mann–Whitney tests) analyses. Pearson’s correlation test was performed on OTUs and VOCs matrix by SPSS (version 20) software.

## 3. Results

### 3.1. Nutritional Assessment and Biochemical Analyses

Twenty patients with a diagnosis of vascular disease (5 patients), subarachnoid haemorrhage and head trauma (6 patients), stroke (6 patients) and aortic dissection complicated by coma (3 patients) were recruited and randomly assigned to the IG group (11 patients) or CG group (9 patients). All patients were completely dysphagic and were fed exclusively through EN.

Analysing nutritional measurements and haematochemical values, no difference between T_0_ and T_1_ for both IG and CG groups was observed (Table 1).

### 3.2. Constipation and Stool Consistency

**Figure 1:**
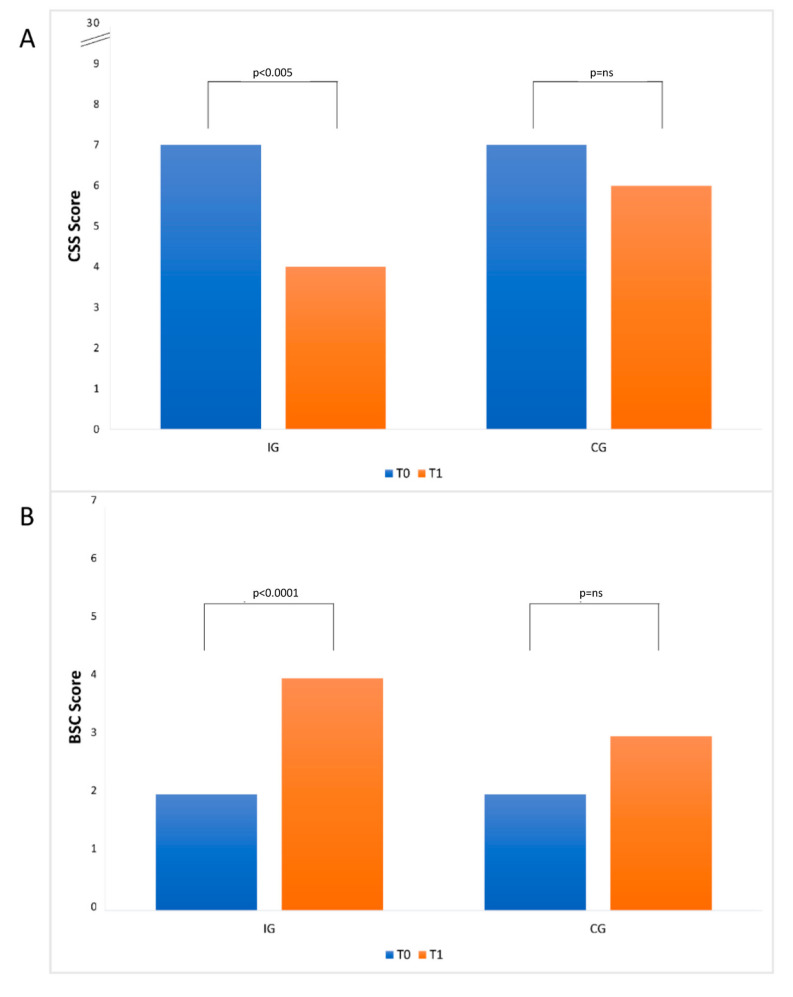
Histograms of constipation scoring system (CSS) (**A**) and Bristol Stool Chart (BSC) (**B**) for IG and CG groups at T_0_ and T_1_. Panel A: The *y*-axis reports a portion of the score’s scale (from 0 (absence of constipation) to 30 (maximum grade of constipation)) at T_0_ and T_1_; panel B: the *y*-axis reports a portion of the score’s scale (from 1 (maximum grade of stool hardness) to 7 (entirely liquid stool)) at T_0_ and T_1_. In both histograms, the median values of each score are reported.

Comparing the results of the constipation evaluation, the IG group showed at T_1_ a statistical reduction (*p* < 0.005) with respect to the T_0_ point of the CSS value, while the CG group showed a reduction of CSS between T_0_ and T_1_ points not statistically significant (*p*-value = ns; Figure 1, Panel A). Regarding stool consistency, the IG group at T_1_ showed a statistically significant increase (*p* value < 0.0001) of BCS value, while the CG groups showed a not statistically significant increase of the same value (*p*-value = ns; Figure 1, Panel B).

### 3.3. Composition of Intestinal Microbiota at Baseline (T_0_) and after Four Months of Intervention (T_1_)

**Figure 2:**
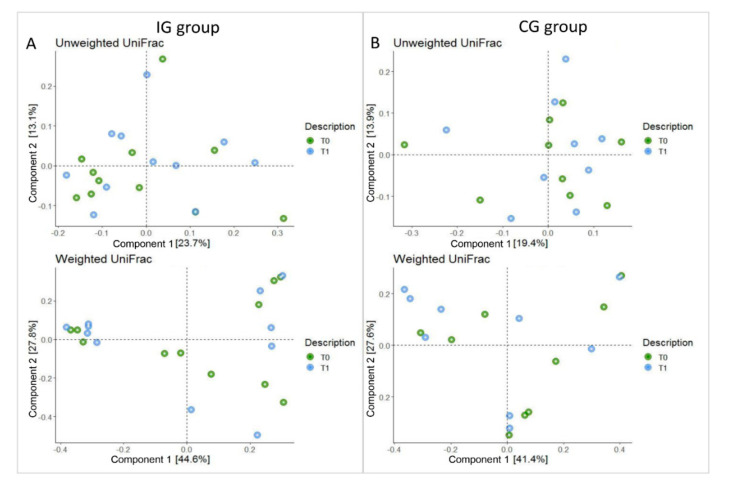
Principal component analysis plot (PCA) of the bacterial communities using UniFrac algorithm. Axes represent the first two components from the principal coordinate (PCo) analysis, based on the phylogenetic distance between operational taxonomic units (OTU) representative sequences. (Panel **A**): UniFrac unweighted PCoA and weighted plots of IG group. (Panel **B**): unweighted and weighted UniFrac PCoA plots of CG group. T_0_ and T_1_ refer to baseline and four months of synbiotics administration, respectively.

Ecological analyses were conducted on the patients’ cohort in order to analyse GM OTUs ecology and global distribution. Beta diversity indices (i.e., weighted and unweighted UniFrac) did no detect any statistically significant differences between T_0_ and T_1_ for the IG and CG groups (Adonis test *p* > 0.05; Figure 2).

**Figure 3:**
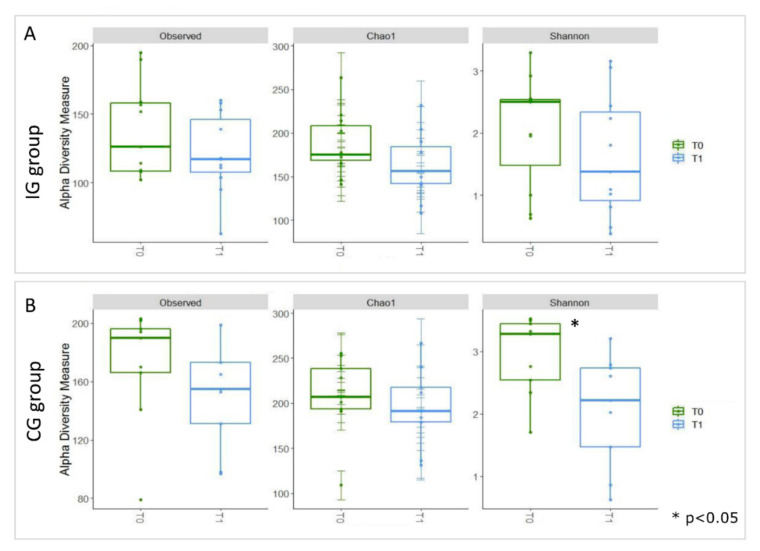
Boxplots representing α-diversity indices. The box plots represent the Observed, Chao1, and Shannon index-es for samples stratified for T_0_ and T_1_ in the IG (**A**) and CG (**B**) groups. The interquartile range is represented by the box, and the line in the box is the median. The whiskers indicate the largest and the lowest data points, respectively, while the dots symbolize outliers. The asterisk * indicates a *p*-value < 0.05.

An alpha diversity analysis, carried out using Observed, Chao1, and Shannon indices, highlighted a general trend of decreasing biodiversity for all patients at T_1_ (Figure 3).

In the CG, the Shannon index highlighted a statistically significant decrease between T_0_ and T_1_.

**Figure 4:**
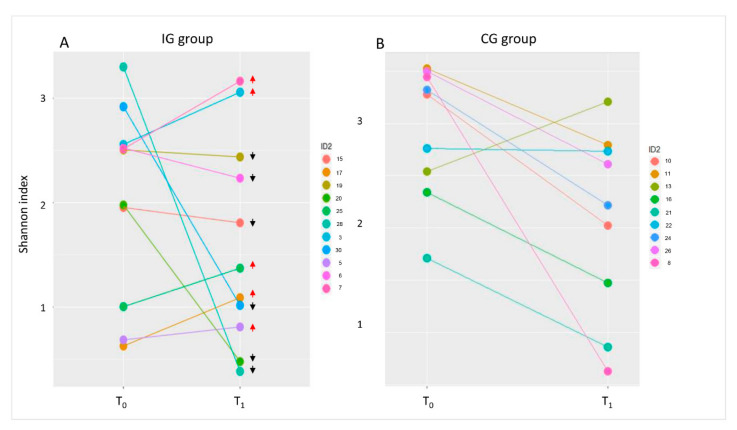
Shannon index trends. Graphs of the Shannon index at T_0_ and T_1_ in IG (**A**) and CG (**B**) groups. Each patient is represented by two coloured dots, one at T_0_ and one at T_1_. Red arrows indicate increases in the Shannon index at T_1_; black arrows indicate decreases in the Shannon index at T_1_.

In order to assess individual differences in the Shannon index, we evaluated for each patient the index variation at T_0_ and T_1_ (Figure 4). It was observed that in about half of the IG patients, 5/11 tended to increase their biodiversity at T_1_.

**Figure 5:**
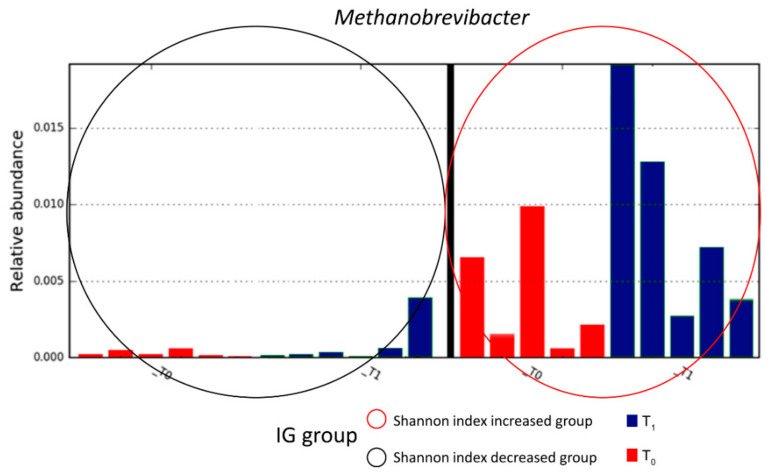
Linear discriminant analysis (LDA) effect size (LEfSe) analysis. Analysis was performed by grouping IG patients on the basis of the Shannon index trend. The red oval indicates the subgroup of patients in which the Shannon index was increased at T_1_, while the black oval indicates the subgroup of patients in which the Shannon index was decreased at T_1_.

Then, IG patients were subdivided on the basis of α-diversity. Through LEfSe analysis, the identification of *Methanobrevibacter* as a microbial biomarker of the IG subgroup, in which Shannon index was increased at T_1_, was clear (Figure 5).

The comparison of gut microbiota composition revealed no statistically significant differences between T_0_ and T_1_. In the CG group, *Faecalibacterium* spp., *Agrobacterium* spp., and *Flavobacterium* spp. decreased at T_1_, while WAL_1855D increased (Supplementary Table S1).

Finally, the relative abundance of genera constituting the Syngut product administered to IG patients was evaluated to observe whether the synbiotic actually affected their relative abundance in faecal samples. *Lactococcus* spp. maintained the same relative abundance between T_0_ and T_1_, while both *Lactobacillus* spp. and *Bifidobacterium* spp. showed a no statistically significant increment in their relative abundance after probiotic administration (T_1_; Supplementary Table S1).

### 3.4. Volatile Metabolome Profile at Baseline (T_0_) and after Four Months of Intervention (T_1_)

By GC-MS/SPME, we identified and quantified 166 VOCs. These molecules were grouped into 17 chemical classes by alcohols (*n* =23), alkenes (*n* =25), alkanes (*n* =22), ketones (*n* =24), esters (*n* =21), acids (*n* =7), amides (*n* =1), phenols (*n* = 7), pyridine (*n* = 7), pyrazine (*n* = 1), indole (*n* = 6), aldehydes (*n* = 15), aromatic hydrocarbons (*n* = 1), furans (*n* = 1), furfural (*n* = 1), terpenes (*n* = 2), and sulphur compounds (*n* = 2).

The metabolic profiles of each sample showed a high variability among subjects.

The raw data matrix was condensed into a 36 metabolite matrix, maintaining metabolites present in at least 15% of the entire set of samples. On this condensed matrix, the Mann–Whitney test of volatile metabolites between T_0_ and T_1_ in CG and IG groups was performed (Supplementary Table S2). The test did not evidence statistically significant differences between groups.

However, short chain fatty acids (SCFAs), particularly propionic and butanoic acids, showed increased levels in IG subject after synbiotic administration. This trend was observed for other metabolites, such as ketones like 2-octanone and 2-pentadecanone.

On the contrary, molecules such as p-cresol, benzaldehyde, and indole showed decreased levels in the intervention group (Supplementary Table S2).

### 3.5. Pearson’s Correlation Test of Targeted Metagenomic and VOCs Data

The Pearson’s correlations amongst genera constituting the Syngut product administered to IG patients were studied. *Lactobacillus* spp., *Bifidobacterium* spp., *Lactococcus* spp., and SCFAs as butanoic and propionic acids, and other OTUs and VOCs highlighted some statistically significant and positive correlations (Supplementary Table S3). Particularly, *Lactobacillus* spp. and *Bifidobacterium* spp. showed a concomitant trend in the positive correlations (*p* ≤ 0.05) with other OTUs (i.e., *Actinomyces*, *Brevibacterium*, *Coprobacillus*, *Corynebacterium*, *Eggerthella*, *Enterococcus*, *Methanosphaera*, *Paludibacter, Pediococcus*, *Peptococcus*, *Pseudoramibacter*, *Eubacterium*, *Pyramidobacter*, *Staphylococcus*, and *Streptococcus*), and VOCs (i.e., 3-Heptanone, 8-Nonen-2-one, Methyl Isobutyl Ketone, and Phenylethyl Alcohol). *Lactococcus* showed positive correlations only with OTUs (i.e., *Agrobacterium*, *Anaerostipes*, *Bacteroides*, *Leuconostoc*, and *Ochrobactrum*). Moreover, the butanoic and propanoic acid also showed a concomitant trend in the positive correlations (*p* ≤ 0.05) with other metabolites (i.e., 1-hexanol, 2-ethyl, 2-heptanone, 2-Tridecanone, butanal, 3-methyl, Cyclopentadecane, and p-Cresol; Supplementary Table S3).

## 4. Discussion

LTHEN patients frequently suffer from abnormal bowel function, which affects their GM and quality of life. Gut bacteria are involved in bowel health; the normal microbiota provides competitive exclusion for potentially pathogenic organisms (e.g., *Clostridium difficile*) and ferments carbohydrates reaching the colon to produce SCFA. Dysbiosis is thought to contribute to many gut problems [39,40,41].

Some studies have shown that the modulation of GM while receiving enteral nutrition can be mostly associated to remarkable change of Bifidobacteria and Lactobacilli. The combination of fibre and probiotics was effective for the treatment of gut dysfunction associated with enteral nutrition. Whelan showed that Bifidobacteria, one of the main GM probiotics, can vary by 1000-fold in patients who are receiving enteral nutrition [42]. Several factors are involved in the pathogenesis of diarrhoea, in which the disruption of GM can play a key role. GM can affect a variety of intestinal functions, such as the maintenance of the integrity of the epithelial barrier and the development of mucosal immunity [43,44]. Meanwhile, GM can also produce a variety of substances, ranging from fatty acids (FAs) [45] and peroxides [46] to highly specific bacteriocins [47], which can inhibit or kill other potentially pathogenic bacteria [41].

The four different probiotic strains that compose the Syngut were selected for their demonstrated ability to survive the gastrointestinal tract and capability to induce strain-specific beneficial effects, such as strengthening the gut barrier function after immunological-induced stress and significantly inhibiting interleukin (IL)-4, IL-5, and IL-13, in addition to stimulating IL-10 levels, which has an immunomodulatory effect [48].

Moreover, these bacterial strains are able to produce significant amounts of β-galactosidase, thus facilitating lactose digestion. This represents a promising approach for the management of patients with lactose intolerance, which is by far the most frequent food intolerance in the population [49].

In an observational study, the efficacy of this synbiotic formulation was demonstrated in the treatment of adult subjects affected by irritable bowel syndrome (IBS) [50]. In particular, the improvement of IBS symptoms and the reduction of faecal calprotectin was reported after two months of treatment in respect to baseline [50].

The synbiotic formula used in this study, was enriched by the prebiotic inulin. Prebiotics are defined as non-digestible food ingredients that beneficially affect the host by selectively stimulating the growth and/or activity of one or a limited number of bacteria in the colon [51]. The effect of inulin is to selectively stimulate the growth and/or activity of beneficial bacteria, such as *Lactobacillus* and *Bifidobacterium* species. It has been used for decades in research and is on the market both as a prebiotic as well as in combination with probiotics, which make a synbiotic [48].

Early literature suggests that using synbiotics may be more effective for restoring the GM compared with probiotics alone, but controversies around the effect on clinical outcomes remain [52,53,54].

For long-term patients, evidence from outpatient studies indicates that dietary fibre may have a strong metabolic immunomodulatory effect in chronic inflammatory diseases, and the use of fibre-enriched enteral formula as part of their nutritional regimen is advisable. In critically ill patients, the small number of available studies seems to indicate at least safety in this high-risk population [55,56,57].

Moreover, in LTHEN patients, constipation is often present. In this setting, constipation is multi factorial, in part due to a lack of mobility, and in part to the chronic ingestion of a liquid diet, but especially due to spastic condition. Constipation not only worsens nutritional status, but also the gastrointestinal symptoms [20]. The use of Syngut in patients in EN improves the intestinal function and regularity. In fact, in the IG group there is an improvement of constipation, the main complication of long-term enteral nutrition (LTEN), and in the consistency of stool between T_0_ and T_1_; it is possible to assert that the optimal time of action of the synbiotic is four months. This hypothesis is confirmed by other studies [24,25], but they are conducted separately with probiotics or prebiotics.

This pilot study, for the first time in literature, evaluates the effect of the administration of synbiotic in LTHEN patients investigating the patient intestinal function, the GM modification, and its functional activity.

Concerning other outcomes (i.e., quality of life and haematochemical results), the use of synbiotics is not responsible for any improvement; in fact, there were no differences between T_0_ and T_1_ for IG and CG. This, in accordance with other studies [58], may be due to there being no differences in the enteral feeding. Four months, the time period in which the synbiotic Syngut was evaluated, is a relatively short time to verify chemical–biochemical changes, and there are a small number of samples under examination.

IG patients presented increased biodiversity compared to CG. This result is also confirmed by the several synergistic positive correlations that the Syngut components, *Lactobacillus* spp. And *Bifidobacterium* spp., have established with other GM bacteria. Moreover, *Methanobrevibacter* was linked to higher GM biodiversity in the synbiotic group.

*Methanobrevibacter* belongs to the Archaea kingdom and Euryarchaeota phylum. It is present in considerable proportions in the gut [59]. This microorganism is a methanogen and plays a key role in gut microbial metabolism of hydrogen [60], by removing hydrogen gas and producing methane. Removal of hydrogen gas affects bacterial fermentation and energy harvesting [61]. In particular, some bacterial components of GM, from the fermentation of fibre and inulin, produce SCFAs that are consumed by methanogenic bacteria to generate methane [60]. From our results, we can speculate that inulin, contained in the synbiotic formula, stimulates the growth of probiotic biomass, enhancing the levels of all end-products of their metabolism, like the release of SCFAs as evidenced by our metabolomics analysis.

This event could promote the growth of methanogenic bacteria, like *Methanobrevibacter*, which consume SCFAs to generate methane. However, larger studies on the effect of inulin on *Methanobrevibacter* metabolism are required to confirm our results.

Moreover, in our study, beneficial molecules such as propionic and butanoic acids increased, and potentially harmful molecules such as p-cresol, benzaldehyde, and indoles decreased after synbiotic administration. Particularly, these latter negative biomarkers have been usually associated with intestinal dysbiosis conditions [62,63].

Taken together, our results could present an indication to clinicians to consider synbiotic administration in LTHEN patients.

## 5. Conclusions

In conclusion, the use of Syngut in LHTEN patients for four months ameliorated constipation and the consistency of stool. Although the synbiotic intake seems to not massively affect the GM composition and its functional activity, it assisted with the improvement of microbiota richness, especially in patients characterized by the presence of *Methanobrevibacter*. This microorganism seems to benefit from synbiotic intake, also correlated to the increment of GM richness.

## Figures and Tables

**Table 1:** Clinical and nutritional feature comparison between T_0_ and T_1_ for both IG and CG groups, computed by Mann–Whitney test. Each parameter is reported as average ± standard deviation.

Blood Variables	IG T_0_	IG T_1_	*p*-Value	CG T_0_	CG T_1_	*p*-Value
Glycemia (g/dL)	81 ± 30	79 ± 32	0.11	89 ± 22	87 ± 32	0.10
Total Protein (g/dL)	6.6 ± 0.50	6.3 ± 0.50	0.14	6.7 ± 0.50	6.6 ± 0.50	0.12
Albumin (g/dL)	3.7 ± 0.30	3.7 ± 0.30	0.17	3.7 ± 0.20	3.8 ± 0.30	0.11
Transferrin (g/dL)	205 ± 75	209 ± 39	0.17	211 ± 78	212 ± 42	0.16
Cholesterol (mg/dL)	153 ± 21	148 ± 34	0.12	160 ± 44	157 ± 50	0.16
Triglycerides (mg/dL)	75 ± 20	82 ± 40	0.11	86 ± 18	91 ± 22	0.12
Haemoglobin (g/dL)	13.2 ± 1.20	13.4 ± 1.30	0.13	13.40 ± 2.10	13 ± 2	0.14
Vitamin B 12 (ng/L)	567 ± 204	547 ± 198	0.10	588 ± 334	573 ± 292	0.11
Folic Acid (ng/L)	15.6 ± 4.20	15.3 ± 3.90	0.13	16.30 ± 3.20	17.3 ± 4.20	0.15
**Enteral Nutrition Intake**						
Protein (g)	62 ± 16	61 ± 16	0.10	64 ± 17	63 ± 12	0.10
Lipid (g)	60 ± 14	58 ± 11	0.12	61 ± 5	60 ± 11	012
Carbohydrates (g)	180 ± 37	173 ± 21	0.11	185 ± 188	182 ± 28	0.11
Energy (kcal)	1508 ± 212	1457 ± 188	0.11	1545 ± 177	1520 ± 161	0.10
Fibre (g)	18 ± 4	17 ± 2	0.10	15 ± 4	16 ± 3	0.10

## Data Availability

All Illumina sequencing raw reads and associated metadata are available at NCBI: Bioprojects PRJNA664661.

## Supplementary Materials

The following are available online at https://www.mdpi.com/2072-6643/13/1/87/s1, Table S1: Mann–Whitney test of bacterial taxa between T_0_ and T_1_ in CG and IG groups. Average values of relative abundances of each OTU and the *p* values of each comparison were reported. Table S2. Mann-Whitney test of volatile metabolites between T_0_ and T_1_ in CG and IG groups. Median values of levels of each metabolite and the *p* values of each comparison were reported. Table S3. Pearson’s correlation analysis amongst genera constituting the Syngut product administered to IG patients (i.e., *Lactobacillus* spp., *Bifidobacterium* spp., *Lactococcus* spp.,) and SCFAs (i.e., butanoic and propionic acids). Selected Pearson’s rho values on the base of *p* value (*p* < 0.05) were reported. In green Pearson’s rho and *p* values statistically relevant.
