# Supplementary material for: Effects of a Synbiotic Formula on Functional Bowel Disorders and Gut Microbiota Profile during Long-Term Home Enteral Nutrition (LTHEN): A Pilot Study
Source: Nutrients. 2020 Dec 29;13(1):87. doi: 10.3390/nu13010087 (PMC7824736; doi:10.3390/nu13010087)
Supplement: Supplementary file 1 [file nutrients-13-00087-s001.pdf]

Supplementary materials

Supplementary Table S1. Mann–Whitney test of bacterial taxa between T0 and T1 in CG and IG groups. Average values of relative abundances of each OTU and the p values of each comparison were reported.

**Bacterial Taxa**		**CG**			**IG**		
**Phylum**	**Genus**	**T0**	**T1**	***p-value***	**T0**	**T1**	***p-value***
[Thermi]	*Deinococcus*	0.002	0.000	0.374	0.000	0.000	NA
Actinobacteria	*Actinobaculum*	0.002	0.001	1.000	0.019	0.000	0.242
Actinobacteria	*Actinomyces*	0.674	0.137	0.920	0.643	0.060	0.909
Actinobacteria	*Adlercreutzia*	0.000	0.000	NA	0.000	0.000	NA
Actinobacteria	*Atopobium*	0.012	0.000	0.077	0.005	0.000	0.167
Actinobacteria	*Bifidobacterium*	12.497	8.249	0.190	6.062	6.450	0.847
Actinobacteria	*Brevibacterium*	0.016	0.007	0.332	0.304	0.044	0.582
Actinobacteria	*Collinsella*	2.629	5.768	0.508	1.783	0.161	0.742
Actinobacteria	*Corynebacterium*	1.537	0.034	1.000	0.121	0.013	0.662
Actinobacteria	*Eggerthella*	3.860	1.012	0.258	0.340	0.522	0.669
Actinobacteria	*Mobiluncus*	0.002	0.000	0.374	0.000	0.001	0.363
Actinobacteria	*N09*	0.000	0.000	NA	0.000	0.000	0.363
Actinobacteria	*Propionibacterium*	0.013	0.009	0.258	0.002	0.003	0.660
Actinobacteria	*Pseudoclavibacter*	0.000	0.001	0.374	0.000	0.000	NA
Actinobacteria	*Slackia*	0.005	0.003	1.000	0.001	0.000	0.363
Bacteroidetes	5-7N15	0.000	0.000	NA	0.000	0.000	NA
Bacteroidetes	*Bacteroides*	12.173	14.273	0.387	11.424	22.830	0.652
Bacteroidetes	*Butyricimonas*	0.436	0.668	0.859	0.225	0.204	0.461
Bacteroidetes	*Cloacibacterium*	0.002	0.000	0.374	0.000	0.000	NA
Bacteroidetes	*Dysgonomonas*	0.000	0.000	NA	0.003	0.009	1.000
Bacteroidetes	*Flavobacterium*	0.039	0.000	0.005	0.006	0.000	0.056
Bacteroidetes	*Flectobacillus*	0.000	0.000	NA	0.000	0.000	NA
Bacteroidetes	*Fluviicola*	0.001	0.000	0.374	0.002	0.000	0.363
Bacteroidetes	*Odoribacter*	0.256	0.278	0.859	0.911	0.135	0.893
Bacteroidetes	*Paludibacter*	0.000	0.000	NA	0.000	0.005	0.363

Bacteroidetes	*Parabacteroides*	6.754	2.054	0.605	1.692	1.449	0.577
Bacteroidetes	*Paraprevotella*	0.102	0.049	1.000	0.121	0.042	0.336
Bacteroidetes	*Porphyromonas*	0.303	0.008	0.911	0.001	0.000	0.363
Bacteroidetes	*Prevotella*	0.030	0.026	0.763	0.033	0.052	0.730
Chlamydiae	*Candidatus Rhabdochlamydia*	0.012	0.000	0.169	0.000	0.000	NA
Euryarchaeota	*Methanobacterium*	0.027	0.003	1.000	0.000	0.000	NA
Euryarchaeota	*Methanobrevibacter*	7.012	9.969	1.000	0.738	0.594	0.818
Euryarchaeota	*Methanosphaera*	0.078	0.031	1.000	0.000	0.001	0.167
Firmicutes	*[Eubacterium]*	0.570	0.165	0.791	3.920	2.287	0.843
Firmicutes	*[Ruminococcus]*	1.513	3.098	0.863	2.085	1.160	0.332
Firmicutes	*Acidaminococcus*	0.096	0.090	0.920	0.689	0.022	0.362
Firmicutes	*Anaerococcus*	0.005	0.005	1.000	0.011	0.000	0.167
Firmicutes	*Anaerofustis*	0.003	0.020	0.275	0.001	0.001	1.000
Firmicutes	*Anaerostipes*	0.005	0.001	0.275	0.000	0.000	0.363
Firmicutes	*Anaerotruncus*	1.199	1.267	1.000	0.535	0.209	0.468
Firmicutes	*Bacillus*	0.021	0.000	0.374	0.000	0.000	NA
Firmicutes	*Blautia*	2.350	0.194	0.387	5.702	6.464	0.797
Firmicutes	*Bulleidia*	0.021	0.006	0.497	0.001	0.000	1.000
Firmicutes	cc_115	0.001	0.001	1.000	0.004	0.011	0.592
Firmicutes	*Christensenella*	1.235	0.144	0.212	0.172	0.102	0.894
Firmicutes	*Clostridium*	0.050	0.030	0.115	0.004	0.003	0.721
Firmicutes	*Coprobacillus*	0.067	0.083	1.000	0.030	0.006	0.531
Firmicutes	*Coprococcus*	1.071	0.338	0.297	0.383	0.430	0.974
Firmicutes	*Dehalobacterium*	0.057	0.026	0.579	0.014	0.008	1.000
Firmicutes	*Desulfitobacter*	0.000	0.003	0.374	0.004	0.000	0.363
Firmicutes	*Dialister*	0.002	0.012	0.655	0.475	0.119	0.947
Firmicutes	*Dorea*	0.947	5.042	0.387	0.658	0.710	0.870
Firmicutes	*Enterococcus*	2.327	1.207	0.605	3.353	0.566	0.307
Firmicutes	*Epulopiscium*	0.000	0.003	0.169	0.000	0.000	NA
Firmicutes	*Facklamia*	0.008	0.006	1.000	0.000	0.000	NA
Firmicutes	*Faecalibacterium*	0.025	0.007	0.032	0.033	0.012	0.756

Firmicutes	*Finegoldia*	0.002	0.020	0.587	0.017	0.001	0.474
Firmicutes	*Geobacillus*	0.001	0.000	0.374	0.000	0.000	NA
Firmicutes	*Holdemania*	0.023	0.009	0.823	0.033	0.013	0.658
Firmicutes	*Jeotgalicoccus*	0.000	0.034	0.374	0.000	0.000	NA
Firmicutes	*Lachnospira*	0.001	0.000	0.374	0.000	0.000	NA
Firmicutes	*Lactobacillus*	0.416	0.930	0.532	0.880	0.940	0.286
Firmicutes	*Lactococcus*	0.002	0.016	0.737	0.013	0.014	0.660
Firmicutes	*Leuconostoc*	0.009	0.001	1.000	0.010	0.008	0.961
Firmicutes	*Megasphaera*	0.255	0.029	0.396	1.969	0.387	0.840
Firmicutes	*Mitsuokella*	0.033	0.003	1.000	0.000	0.000	NA
Firmicutes	*Mogibacterium*	0.013	0.000	0.374	0.000	0.000	NA
Firmicutes	*Oscillospira*	5.253	1.097	0.222	2.022	3.260	0.797
Firmicutes	p-75-a5	0.010	0.006	1.000	0.000	0.000	NA
Firmicutes	*Parvimonas*	0.013	0.002	0.587	0.007	0.001	1.000
Firmicutes	*Pediococcus*	0.010	0.000	0.374	0.000	0.113	0.363
Firmicutes	*Peptococcus*	0.087	0.001	0.225	0.026	0.269	0.849
Firmicutes	*Peptoniphilus*	0.043	0.042	0.763	0.019	0.000	0.363
Firmicutes	*Peptostreptococcus*	0.000	0.003	0.169	0.000	0.000	0.363
Firmicutes	ph2	0.000	0.002	0.374	0.000	0.000	NA
Firmicutes	*Phascolarctobacterium*	0.180	0.274	1.000	1.792	4.858	0.679
Firmicutes	*Pseudoramibacter_Eubacterium*	5.568	0.736	0.330	0.220	0.169	0.322
Firmicutes	*Roseburia*	0.010	0.003	0.160	0.006	0.004	0.943
Firmicutes	*Ruminococcus*	6.024	1.448	0.190	0.684	0.952	0.797
Firmicutes	SMB53	0.281	0.162	1.000	0.507	0.044	0.535
Firmicutes	*Sporanaerobacter*	0.000	0.000	NA	0.000	0.000	NA
Firmicutes	*Staphylococcus*	0.067	0.003	0.073	0.126	0.007	0.867
Firmicutes	*Streptococcus*	0.198	0.089	0.655	0.101	0.095	0.640
Firmicutes	*Syntrophomonas*	0.000	0.000	NA	0.000	0.000	NA
Firmicutes	*Turicibacter*	0.025	0.002	0.856	0.009	0.001	1.000
Firmicutes	*Veillonella*	0.002	0.007	1.000	0.000	0.003	0.167
Firmicutes	WAL_1855D	0.000	0.013	0.034	0.000	0.000	NA

Fusobacteria	*Fusobacterium*	0.005	0.001	0.683	0.006	0.000	0.167
Planctomycetes	*Planctomyces*	0.003	0.000	0.374	0.000	0.000	NA
Proteobacteria	*Acetobacter*	0.000	0.000	NA	0.000	0.000	NA
Proteobacteria	*Achromobacter*	0.004	0.000	0.374	0.000	0.000	NA
Proteobacteria	*Acinetobacter*	0.033	0.012	0.576	2.237	0.265	0.721
Proteobacteria	*Aggregatibacter*	0.004	0.000	0.374	0.000	0.008	0.363
Proteobacteria	*Agrobacterium*	0.049	0.000	0.034	0.000	0.005	0.078
Proteobacteria	*Alcanivorax*	0.006	0.000	0.169	0.000	0.000	NA
Proteobacteria	*Arcobacter*	0.015	0.000	0.374	0.000	0.000	NA
Proteobacteria	*Bdellovibrio*	0.007	0.000	0.374	0.000	0.000	NA
Proteobacteria	*Bilophila*	0.074	0.045	0.280	0.174	0.085	0.123
Proteobacteria	*Blastomonas*	0.001	0.000	0.374	0.000	0.000	NA
Proteobacteria	*Campylobacter*	0.000	0.000	NA	0.047	0.019	1.000
Proteobacteria	*Citrobacter*	1.153	0.152	0.683	1.903	0.006	0.582
Proteobacteria	*Delftia*	0.015	0.001	0.225	0.000	0.000	NA
Proteobacteria	*Desulfovibrio*	0.396	0.351	0.859	0.830	0.321	0.946
Proteobacteria	*Hyphomicrobium*	0.003	0.000	0.374	0.000	0.000	NA
Proteobacteria	*Lautropia*	0.001	0.000	0.374	0.000	0.000	NA
Proteobacteria	*Methylobacterium*	0.000	0.000	NA	0.000	0.000	NA
Proteobacteria	*Neisseria*	0.000	0.005	0.169	0.000	0.000	0.363
Proteobacteria	*Novosphingobium*	0.001	0.000	0.374	0.001	0.000	0.363
Proteobacteria	*Ochrobactrum*	0.006	0.001	0.118	0.004	0.001	0.531
Proteobacteria	*Oligella*	0.000	0.003	0.374	0.000	0.000	NA
Proteobacteria	*Proteus*	0.000	0.000	NA	0.001	0.000	0.363
Proteobacteria	*Pseudomonas*	0.089	0.012	0.269	0.018	0.002	0.721
Proteobacteria	*Psychrobacter*	0.008	0.000	0.169	0.000	0.002	0.363
Proteobacteria	*Ralstonia*	0.005	0.000	0.374	0.000	0.000	NA
Proteobacteria	*Rhodobacter*	0.010	0.000	0.374	0.000	0.000	NA
Proteobacteria	*Sphingobium*	0.001	0.000	0.374	0.000	0.000	NA
Proteobacteria	*Sphingomonas*	0.023	0.000	0.169	0.003	0.001	1.000
Proteobacteria	*Stenotrophomonas*	0.019	0.011	0.371	0.000	0.000	NA

Proteobacteria	*Sutterella*	0.058	0.007	0.706	0.008	0.001	0.066
Synergistetes	*Pyramidobacter*	0.000	0.001	0.374	0.151	0.006	0.531
Verrucomicrobia	*Akkermansia*	19.369	40.135	0.258	43.567	43.542	1.000
Verrucomicrobia	*Candidatus Xiphinematobacter*	0.020	0.000	0.169	0.001	0.000	0.363
Verrucomicrobia	*Luteolibacter*	0.019	0.000	0.374	0.000	0.000	NA
Verrucomicrobia	*Opitutus*	0.027	0.000	0.169	0.000	0.002	0.363
Verrucomicrobia	*Prosthecobacter*	0.007	0.000	0.374	0.004	0.000	0.363

Supplementary Table S2. Mann-Whitney test of volatile metabolites between T0 and T1 in CG and IG groups. Median values of levels of each metabolite and the p values of each comparison were reported.

	CG			IG		
**volatile metabolites**	**T0**	**T1**	***p-value***	**T0**	**T1**	***p-value***
1-Hexanol, 2-ethyl	0.00	0.00	0.91	7.18	12.42	0.91
1H-Indole, 5-methyl	0.00	0.00	0.76	193.39	0.00	0.61
1-Octene, 3-methyl	0.00	0.00	1.00	13.60	0.00	0.61
1-Pentanol	0.00	0.00	1.00	59.63	0.13	0.61
2-Butanone, 3,3-dimethyl	0.00	0.00	0.76	3.04	0.00	0.61
2-Decanone	0.00	16.43	0.26	0.00	0.00	0.91
2-Heptanone	0.00	0.00	0.76	2.37	11.34	0.76
2-Nonanone	0.00	0.00	0.91	0.00	0.00	0.91
2-Octanone	67.62	83.17	0.26	78.58	155.83	0.26
2-Pentadecanone	0.00	0.00	0.76	79.81	95.32	0.91
2-Tetradecanone	0.00	30.83	0.26	0.00	0.00	0.76
2-Tridecanone	84.84	117.18	0.76	101.01	58.60	0.76
2-Undecanone	27.67	12.21	0.91	21.96	60.35	0.35
3-Heptanone	0.00	0.00	NA	14.52	0.00	0.61
3-Hexanone	25.05	23.54	0.91	0.00	62.41	0.26
5-Hepten-2-one, 6-methyl	0.00	18.19	0.35	0.00	0.00	0.48
8-Nonen-2-one	0.00	0.00	0.61	8.50	0.00	0.91
Benzaldehyde	334.77	946.51	0.35	411.00	687.41	0.48
Butanal, 2-methyl	0.00	0.00	0.61	8.47	0.00	0.76
Butanal, 3-methyl	0.00	0.00	0.76	6.16	20.99	0.61
Butanal, 4-hydroxy-3-methyl	20.23	0.00	0.26	0.00	0.00	0.76
Butanoic acid	0.00	0.00	0.61	47.25	67.80	0.76
Cyclopentadecane	7.42	0.00	1.00	17.32	29.64	0.61
Dodecane	20.59	0.00	0.04	13.26	25.26	0.48
Furfural	0.00	23.97	0.48	14.64	0.00	0.61
Indole	1219.46	4059.69	0.35	1086.13	1084.32	0.91

Indole, 3-methyl	330.04	245.48	0.76	0.00	0.00	0.76
Methyl Isobutyl Ketone	61.24	164.46	0.11	145.52	152.94	0.26
p-Cresol	2452.67	7449.69	0.76	9384.52	12851.27	0.91
Phenol	32.01	0.00	0.61	0.00	130.22	0.35
Phenol, 3-methyl-	88.33	200.67	0.76	41.70	0.00	0.76
Phenylethyl Alcohol	0.00	0.00	0.76	74.56	74.56	1.00
Propanoic acid	0.00	0.00	0.61	0.00	5.10	0.48
Pyridine, 2,4,6-trimethyl	27.33	0.00	0.76	0.00	0.00	0.76
Pyridine, 2-ethyl-4,6-dimethyl	22.90	0.00	0.26	0.00	0.00	NA
Tetradecane	0.00	0.00	0.91	0.00	0.00	0.91

Supplementary Table S3. Pearson's correlation analysis amongst genera constituting the Syngut product administered to IG patients (*i.e.*, *Lactobacillus* spp., *Bifidobacterium* spp., *Lactococcus* spp.,) and SCFAs (*i.e.,* butanoic and propionic acids). Selected Pearson's rho values on the base of p value ($p<0.05$) were reported. In green Pearson’s rho and p values statistically relevant.

Pearson's correlation	Bifidobacterium		Lactobacillus		Lactococcus		Butanoic acid		Propanoic acid	
	Rho value	*p-value*	Rho value	*p-value*	Rho value	*p-value*	Rho value	*p-value*	Rho value	*p-value*
Acidaminococcus	-0.24	0.65	-0.22	0.67	-0.35	0.49	0.73	0.10	**0.92**	**0.01**
Actinomyces	**0.99**	**0.00**	**0.99**	**0.00**	-0.01	0.98	0.43	0.40	0.21	0.68
Agrobacterium	-0.24	0.65	-0.23	0.66	**0.89**	**0.02**	-0.40	0.43	-0.30	0.56
Anaerostipes	-0.24	0.65	-0.23	0.66	**0.89**	**0.02**	-0.40	0.43	-0.30	0.56
Bacteroides	-0.35	0.50	-0.34	0.52	**0.96**	**0.00**	-0.57	0.24	-0.44	0.39
Brevibacterium	**0.99**	**0.00**	**0.99**	**0.00**	-0.01	0.99	0.41	0.42	0.20	0.71
Coprobacillus	-0.24	0.65	-0.22	0.67	-0.35	0.49	0.73	0.10	**0.92**	**0.01**
Coprococcus	**1.00**	**0.00**	**0.99**	**0.00**	-0.03	0.96	0.39	0.45	0.18	0.73
Corynebacterium	**0.97**	**0.00**	**0.97**	**0.00**	0.02	0.96	0.35	0.49	0.15	0.77
Eggerthella	**0.99**	**0.00**	**0.96**	**0.00**	-0.08	0.88	0.35	0.50	0.16	0.77
Enterococcus	**1.00**	**0.00**	**0.99**	**0.00**	-0.02	0.97	0.40	0.44	0.19	0.72
Lactobacillus	**0.99**	**0.00**	**1.00**	NA	-0.06	0.91	0.44	0.39	0.18	0.73
Leuconostoc	-0.25	0.63	-0.24	0.65	**0.88**	**0.02**	-0.40	0.43	-0.32	0.54
Methanosphaera	**0.99**	**0.00**	**0.99**	**0.00**	-0.01	0.99	0.41	0.42	0.20	0.71
Ochrobactrum	-0.24	0.65	-0.23	0.66	**0.89**	**0.02**	-0.40	0.43	-0.30	0.56
Paludibacter	**0.99**	**0.00**	**0.99**	**0.00**	-0.01	0.99	0.41	0.42	0.20	0.71
Pediococcus	**0.99**	**0.00**	**0.99**	**0.00**	-0.01	0.99	0.41	0.42	0.20	0.71
Peptococcus	**0.99**	**0.00**	**0.99**	**0.00**	0.01	0.99	0.39	0.44	0.18	0.73
Pseudoramibacter_Eubacterium	**0.99**	**0.00**	**0.99**	**0.00**	-0.01	0.98	0.41	0.43	0.18	0.73
Pyramidobacter	**0.99**	**0.00**	**0.99**	**0.00**	0.07	0.90	0.39	0.45	0.18	0.74
Staphylococcus	**0.96**	**0.00**	**0.96**	**0.00**	0.04	0.95	0.33	0.53	0.13	0.80
Streptococcus	**1.00**	**0.00**	**0.99**	**0.00**	-0.02	0.97	0.42	0.40	0.21	0.69
1-Hexanol, 2-ethyl	-0.01	0.98	0.04	0.95	-0.49	0.33	**0.91**	**0.01**	**0.96**	**0.00**
1-Octene, 3-methyl	**0.99**	**0.00**	**0.99**	**0.00**	-0.01	0.99	0.41	0.42	0.20	0.71
2-Butanone, 3,3-dimethyl	**0.92**	**0.01**	**0.96**	**0.00**	-0.16	0.77	0.44	0.39	0.10	0.85

2-Heptanone	0.11	0.83	0.19	0.72	-0.57	0.24	**0.93**	**0.01**	**0.84**	**0.04**
2-Pentadecanone	0.59	0.22	0.65	0.16	-0.46	0.36	**0.95**	**0.00**	0.76	0.08
2-Tetradecanone	-0.24	0.65	-0.22	0.67	-0.35	0.49	0.73	0.10	**0.92**	**0.01**
2-Tridecanone	0.30	0.57	0.34	0.52	-0.44	0.38	**0.98**	**0.00**	**0.98**	**0.00**
2-Undecanone	0.02	0.97	-0.02	0.97	-0.60	0.21	0.71	0.11	**0.86**	**0.03**
3-Heptanone	**0.96**	**0.00**	**0.99**	**0.00**	-0.11	0.84	0.44	0.39	0.14	0.79
8-Nonen-2-one	**0.81**	**0.05**	**0.87**	**0.02**	-0.22	0.67	0.42	0.41	0.05	0.93
Benzaldehyde	0.75	0.09	0.75	0.09	-0.34	0.50	**0.87**	**0.03**	0.76	0.08
Butanal, 3-methyl	0.09	0.86	0.13	0.81	-0.09	0.87	**0.84**	**0.04**	**0.93**	**0.01**
Butanoic acid	0.39	0.45	0.44	0.39	-0.48	0.34	**1.00**	NA	**0.92**	**0.01**
Cyclopentadecane	-0.14	0.80	-0.11	0.83	-0.40	0.44	**0.81**	**0.05**	**0.96**	**0.00**
Furfural	-0.24	0.65	-0.22	0.67	-0.35	0.49	0.73	0.10	**0.92**	**0.01**
Indole	-0.25	0.64	-0.23	0.66	-0.28	0.60	0.72	0.11	**0.91**	**0.01**
Methyl Isobutyl Ketone	**0.99**	**0.00**	**0.96**	**0.00**	-0.04	0.94	0.27	0.61	0.05	0.92
p-Cresol	0.21	0.69	0.25	0.64	-0.49	0.32	**0.97**	**0.00**	**0.98**	**0.00**
Phenol, 3-methyl-	-0.26	0.61	-0.25	0.64	-0.34	0.51	0.71	0.12	**0.90**	**0.01**
Phenylethyl Alcohol	**0.81**	0.05	**0.87**	**0.03**	0.11	0.83	0.30	0.56	-0.04	0.95
Propanoic acid	0.16	0.76	0.18	0.73	-0.39	0.45	**0.92**	**0.01**	**1.00**	NA
Tetradecane	-0.24	0.65	-0.22	0.67	-0.35	0.49	0.73	0.10	**0.92**	**0.01**
